# Supplementary material for: Unifying equivalences across unsupervised learning, network science, and imaging/network neuroscience
Source: ArXiv. 2025 Aug 12:arXiv:2508.10045v1. Preprint. [Version 1] (PMC12364054)
Supplement: Supplement 1 [file NIHPP2508.10045v1-supplement-1.pdf]

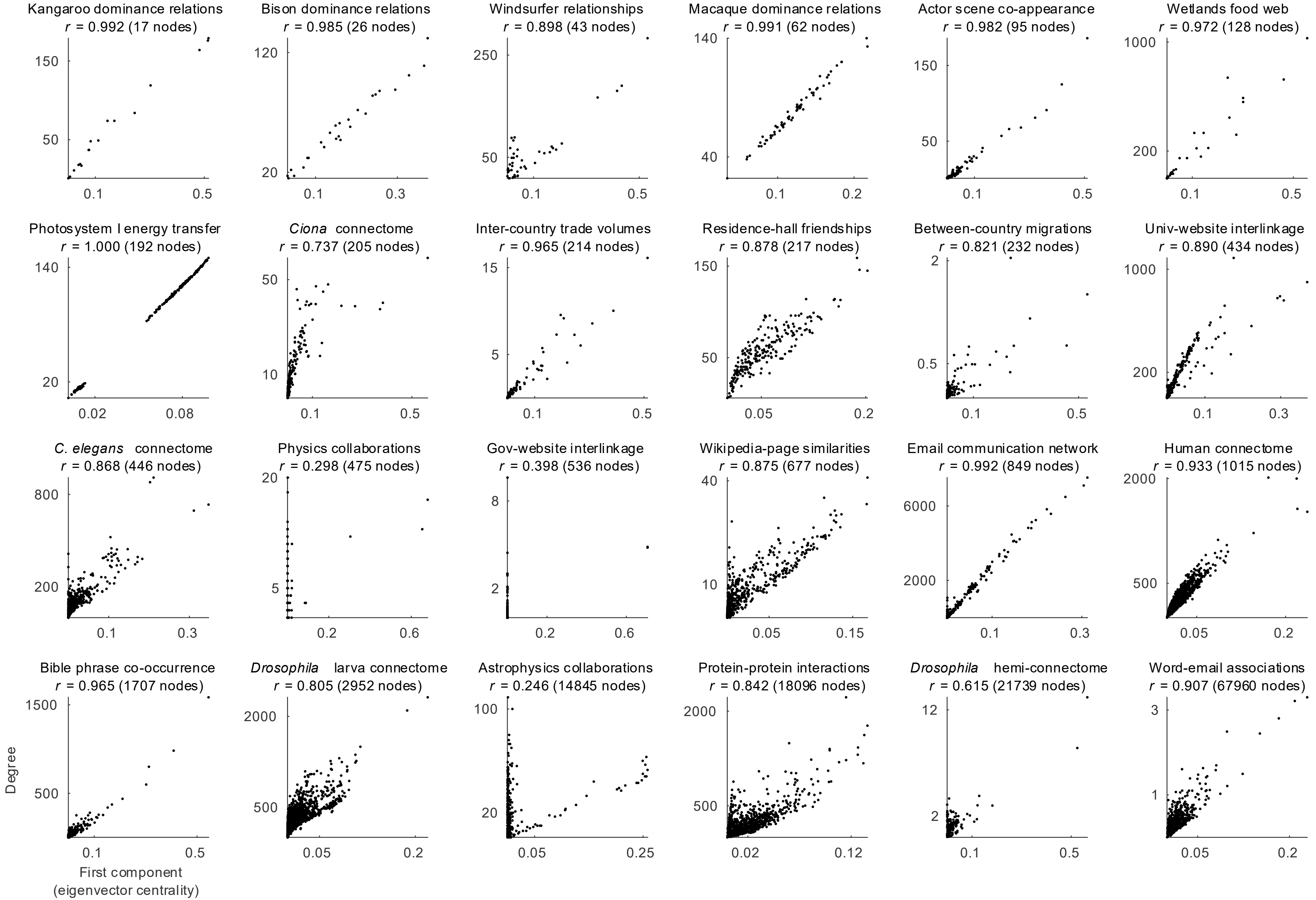


**Figure S1. First component and degree in diverse networks.**

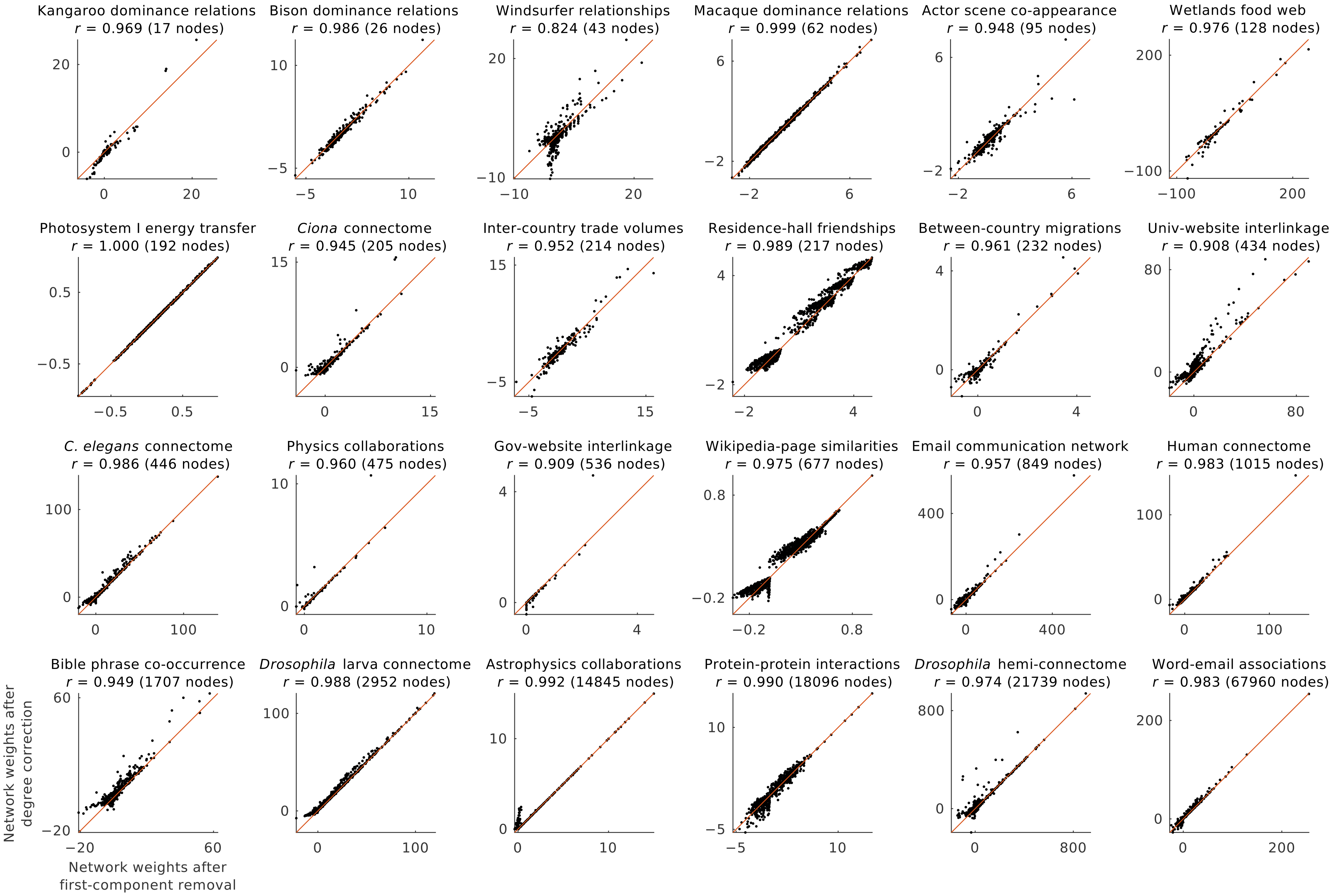


**Figure S2. First-component removal and degree correction in diverse networks.**

## Figure S1. First component and degree in diverse networks.

Comparison of the first component and degree in networks from the Netzschleuder catalogue (Peixoto, 2020). From each dataset in this catalogue, we chose one directed or undirected, non-negatively weighted, and non-temporal network with the largest maximal connected component that satisfied (mean binary degree) > 10 to avoid extreme sparseness and (squared coefficient of variation of binary degree) < 10 to avoid extreme degree heterogeneity (Broido and Clauset, 2019). We analyzed the maximal connected components of these networks. We symmetrized all directed networks by adopting the maximum value for pairs of directed connection weights. We excluded datasets that lacked networks with these criteria, as well as the two outlier datasets that each had tens of thousands of networks (human_brains and open-streetmap). For networks with more than 10,000 nodes, we analyzed ~50,000,000 connections, periodically sampled from 1 to the total number of connections. Finally, we ordered the plots by network size.

Here is a full list of dataset/network abbreviations, in alphabetical order: arxiv_collab/astro-ph-1999 (Newman, 2001); bag_of_words/enron (Newman, 2008); bible_nouns (Harrison and Römhild, 2008); bison (Lott, 1979); budapest_connectome/all_20k (Szalkai et al., 2017); celegans_2019/hermaphrodite_chemical (Cook et al., 2019); cintestinalis (Ryan et al., 2016); dnc (Kunegis, 2013); fao_trade (De Domenico et al., 2015b); fly_hemibrain (Scheffer et al., 2020); fly_larva (Winding et al., 2023); foodweb_baywet (Ulanowicz and DeAngelis, 2005); kangaroo (Grant, 1973); macaques (Takahata, 1991); mist/ppi_human (Hu et al., 2018); moviegalaxies/261 (Kaminski et al., 2012); physics_collab/pierreAuger (De Domenico et al., 2015a); psi (Montepietra et al., 2020); residence_hall (Freeman et al., 1998); un_migrations (UN Population Division, 2015); us_agencies/virginia (Kosack et al., 2018); webkb/webkb_washington_cocite (Slattery and Craven, 1998); wiki_science (Calderone, 2020); windsurfers (Freeman et al., 1988).

## Figure S2. First-component removal and degree correction in diverse networks.

Comparison of network weights after first-component removal and network weights after degree correction in networks from the Netzschleuder catalogue. See Figure S1 for methodological details.

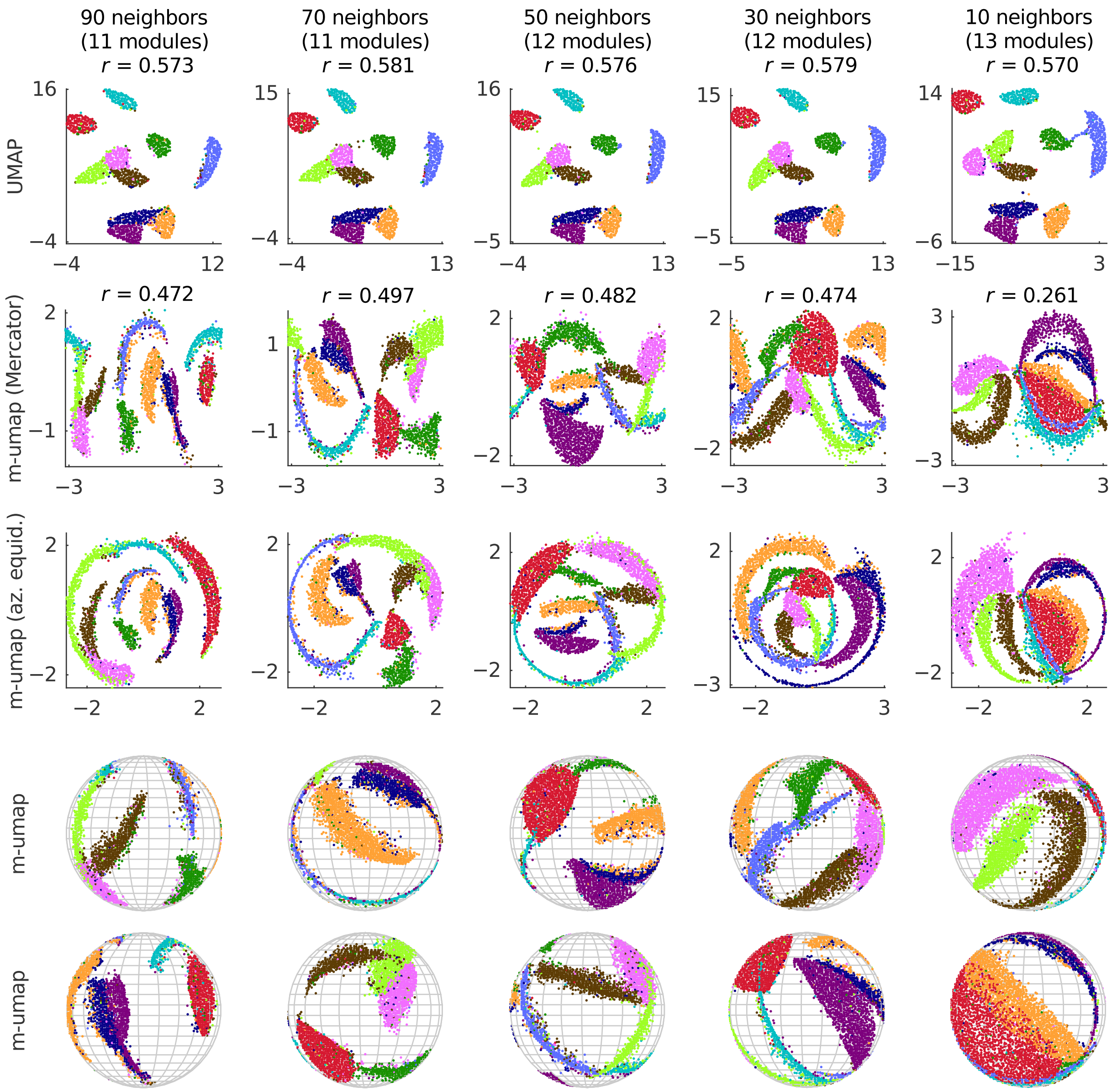


## Figure S3. Performance of UMAP and m-umap on the MNIST handwritten-digit dataset.

UMAP and m-umap embeddings constructed from the symmetric $\kappa$-nearest-neighbor network of the 70,000 images of handwritten digits, using cosine-similarity distance. Columns show results across a range of $\kappa$-nearest neighbors. Colors in all panels denote the ground-truth MNIST labels for the individual digits. $r$ values represent Pearson correlation coefficients between distances of nodes to each MNIST-label centroid, in native and embedding space.

**Row 1.** Two-dimensional UMAP embeddings.

**Row 2.** Mercator ("classic map") projection of spherical m-umap embeddings onto a plane.

**Row 3.** Azimuthal equidistant ("UN flag") projection of spherical m-umap embeddings onto a plane.

**Rows 4–5.** Hemispheric views of spherical m-umap embeddings.
